# Chemical Composition and Antioxidant/Antimicrobial Activities in Supercritical Carbon Dioxide Fluid Extract of *Gloiopeltis tenax*

**DOI:** 10.3390/md10122634

**Published:** 2012-11-22

**Authors:** Jiaojiao Zheng, Yicun Chen, Fen Yao, Weizhou Chen, Ganggang Shi

**Affiliations:** 1 Department of Pharmacology, Shantou University Medical College, Shantou 515041, China; Email: zheng-jiaojiao@163.com (J.Z.); chenyicun@yeah.net (Y.C.); g_fyao2000@yahoo.com.cn (F.Y.); 2 Marine Biology Institute, Shantou University, Shantou 515063, China; Email: wzchen@stu.edu.cn; 3 Department of Cardiovascular Diseases, First Affiliated Hospital, Shantou University Medical College, Shantou 515041, China

**Keywords:** *Gloiopeltis tenax*, GC-MS, CO_2_-SFE, antioxidant activity, antimicrobial activity

## Abstract

*Gloiopeltis tenax* (*G. tenax*) is widely distributed along the Chinese coastal areas and is commonly used in the treatment of diarrhea and colitis. This study aimed at investigating the bioactivities of the volatile constituents in *G. tenax*. We extracted the essential constituents of *G. tenax* by supercritical carbon dioxide extraction (CO_2_-SFE), then identified and analyzed the constituents by gas chromatography-mass spectrometry (GC-MS). In total, 30 components were identified in the *G. tenax* extract. The components showed remarkable antioxidant activity (radical scavenging activity of 2,2-diphenyl-1-picrylhydrazyl (DPPH)), lipid peroxidation inhibition capacity (in a β-carotene/linoleic acid-coupled oxidation reaction), and hydroxyl radical-scavenging activity (by deoxyribose degradation by iron-dependent hydroxyl radical), compared to butylated hydroxytoluene. In microdilution assays, *G. tenax* extracts showed a moderate inhibitory effects on *Staphyloccocus aureus* (minimum inhibitory concentration (MIC) = 3.9 mg/mL), *Enterococcus faecalis* (7.8 mg/mL), *Pseudomonas aeruginosa* (15.6 mg/mL), and *Escherichia coli* (3.9 mg/mL). Antioxidant and antimicrobial activities of *G. tenax* were related to the active chemical composition. These results suggest that the CO_2_-SFE extract from *G. tenax* has potential to be used as a natural antioxidant and antimicrobial agent in food processing.

## 1. Introduction

*Gloiopeltis tenax* is an annual red alga that belongs to the *Rhodophyta phylum*, the *Florideophyeeae* class, the *Cryptonemiales* order, and the *Endocladiaceae GloioPeltis J. Agardh* family. *Gloiopeltis* consist of seven species, distributed in the temperate waters of the North Pacific coast or slightly to the north-south extension [1]. In China, there are only *Gloiopeltis tenax* and *Gloiopeltis furcata*, edible species that have been used for medicinal purposes, and also as a precious resource of marine algae [2]. 

Antioxidants have multiple functions in biological systems, including defense against oxidative damage and participation in the major cell signaling pathways. One principal cellular function of antioxidants is to prevent damage caused by the action of reactive oxygen species (ROS) [3]. ROS are responsible for aging [4], and excessive ROS have been implicated in the cause of various human diseases, such as diabetes [5], neurodegenerative disease [6] and cancer [7]. Different studies show that antioxidant substances that scavenge free radicals play an important role in the prevention of free radical-induced diseases. Several synthetic antioxidants, such as butylated hydroxyanisole (BHA), butylated hydroxytoluene (BHT), and tert-butyhydroquinone (TBHQ), are commercially available and currently used. However, concerns about their safety and toxicity are hindering their use by the food industry [8,9]. In addition, due to the occurrence of resistance to antimicrobials and the incidence of infectious diseases, there is a need to search for new antimicrobial compounds that may inhibit microorganisms by different mechanisms than those in current use [10,11]. Therefore, research on alternative antioxidants and antimicrobials from natural origins has drawn increasing attention. Particularly in recent years consumer awareness of food quality and safety issues has significantly improved. 

Great effort has been focused on, e.g., medicinal plants for the extraction of natural and low-cost antioxidants and antimicrobials that can replace synthetic additives. Oxidative stress results from an imbalance between excess prooxidants and depletion of antioxidants. Algae are exposed to large amounts of light and high concentrations of oxygen during their life cycle. This combination favors the generation of free radicals, as well as other powerful oxidizers [12]. It is suggested that the absence of oxidative damage in the structural components of the algae and their stability against adverse conditions are due to the presence of antioxidants. Therefore, algae can be expected to serve as a rich source of antioxidants. Research on *Gloipeltis* algae has mainly concentrated on *G. furcata*, while *G. tenax* has remained little characterized. The reason is that *G. tenax* is further offshore and distributed in lower mid-tidal area, whereas *G. furcata* is distributed in the mid-tidal area. Therefore *G. tenax* has been less accessible than *G. furcata*, and biological yield has been very limited due to difficulty in acquisition.

Many reports have been published highlighting the variety of biological activities of *Gloiopeltis*. For instance, Fang *et al.* [2] identified 18 compounds with anticholinesterase activity from *G. furcata* extracts. With IC50 values ranging from 1.14 to 15.89 μg/mL, these compounds exhibited mild AChE/BchE inhibitory activities. Bae *et al.* [13] found that methanol extracts of *G. furcata* markedly induced G2/M arrest and reduced the viability of HepG2 Cells in a concentration-dependent manner (0–100 μg/mL). Ethyl acetate extracts of *G. furcata* inhibit production of pro-inflammatory mediators with 100 μg/mL [14], and funoran at 0.1% concentration from *G. furcata* strongly inhibited both bacteria causing dental caries and *Streptococcus sobrinus* adsorption to saliva-coated hydroxyapatite more than 50% [15,16]. Polysaccharides from *G. tenax* have been shown to have anti-tumor effects through augmentation of T-helper, T-cytotoxic and NK cells, inhibited the growth of tumors by 36.4%–65.6% at doses of 10 and 50 mg/kg, and prolonged the survival time by 33.3%–79.2% [17]. These results indicate that *Gloiopeltis* extracts play different roles through different mechanisms. We demonstrate that these biological activities are not just related to the water-soluble polysaccharide, but may be connected with fat-soluble ingredients such as essential oils. To the best of our knowledge, there are no previous reports on chemical composition and biological activity of essential oils of *G. tenax*. The aims of this study were to characterize the composition of the *G. tenax* by gas chromatography-mass spectrometry (GC-MS) and physiological activity. The goal was also to test the antioxidant and antibacterial activities of the analyzed the *G. tenax* extract as a potentially new source of biologically active natural products.

## 2. Results and Discussion

### 2.1. Gas Chromatography-Mass Spectrometry (GC-MS) Analysis of CO_2_-SFE Extracts from *G. tenax*

**Figure 1:**
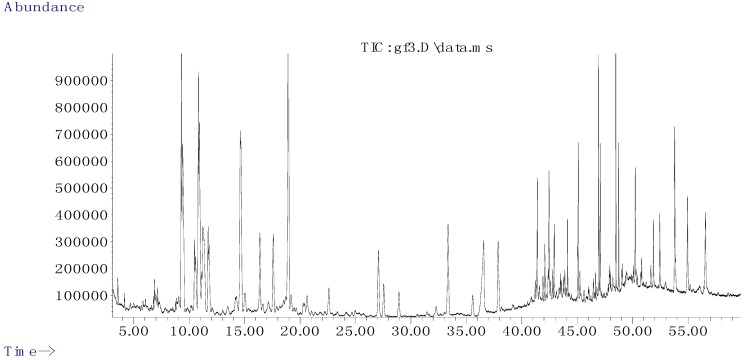
The gas chromatography-mass spectrometry (GC-MS) total icon chromatogram (TIC) of volatile constituents from *G. tenax*.

The ingredient of *G. tenax* were extracted by CO_2_-SFE and analyzed by GC-MS. Chemical composition was separated by programmed temperature gas chromatography, and then individual MS fragments were analyzed. The percentage composition of the extract was computed by normalization to the GC peak areas without using correction factors. Identification of constituents was based on comparison of their Kovats Index (KI) and the MS fragmentation pattern with reference compositions in the database of the NIST Mass Spectral Search Program. The GC-MS total ion chromatogram (TIC) of the *G. tenax* extraction is shown in Figure 1.

Thirty compounds were identified in all of the oils analyzed, twenty-three of which are listed in Table 1. The compounds that are not listed in Table 1 are alkanes. 78% were identified by GC-MS combined with KI. The others were not identified in the database of the NIST Mass Spectral Search Program, possibly because the comparison of the obtained mass spectra with those of reference compounds was too low to enable identification. The identified components include six sesquiterpenes (14.39%), three ketones (5.02%), seven fatty acids and their esters (29.1%), two phenols (1.71%), and three sterols (12.81%) (Table 1).

**Table 1:** The chemical composition of the extract identified by GC-MS and Kovats Index (KI).

NO.	RT ^a^	Constituents	KI ^b^	% ^c^
1	7.890	*p*-hydroxybenzaldehyde	1374.4	0.57
2	9.302	(−)-thujopsene	1437.1	4.68
3	10.484	α-curcumene	1484.0	1.54
4	10.833	α-zingiberene	1497.8	2.98
5	11.285	(+)-cuparene	1511.4	0.28
6	11.322	(−)-β-bisabolene	1512.5	1.00
7	14.703	cedrol	1607.3	3.91
8	16.431	vanillylacetone	1644.8	1.92
9	19.016	*n*-heptadecane	1700.7	10.30
10	23.014	myristic acid	1769.8	2.85
11	27.614	fitone	1842.1	2.53
12	33.382	methhyl hexadecanoate	1927.6	1.32
13	37.460	palmitic acid	1987.3	21.21
14	41.236	linoleic acid	2092.9	0.23
15	41.354	hexadeca-1,4-lactone	2096.5	0.57
16	42.602	*cis*-9-octadecenoic acid	2153.2	0.73
17	43.002	stearic acid	2172.0	0.93
18	46.128	oleamide	2361.5	0.24
19	46.948	2,2′-methylenebis(6-*tert*-butyl-4-methylphenol)	2422.4	1.14
20	48.040	2-monopalmitin	2511.2	1.83
21	52.476	cholesta-4,6-dien-3β-ol	2894.9	6.62
22	56.619	cholesterol	3122.3	5.74
23	58.639	cholesta-3,5-dien-7-one	3196.6	0.45

^a^ Retention times (min); ^b^ Kovats Indexes were calculated from our analyses with respect to a series of *n*-alkenes; ^c^ Percentage of relative amount to total.

Extraction of volatile components using conventional processes, such as solvent extraction, is conducted at high pressure and temperature and is time-consuming. Supercritical fluid extraction (SFE) is a rapid, selective, effective and convenient technique for sample preparation before the analysis of compounds. Supercritical fluids have been used as solvents for a wide variety of applications, such as essential oil extraction, and more than 90% of all analytical SFE is performed with CO_2_. Under conditions of low pressure and low temperature, and no influence of solvent, use of CO_2_-SFE is a good way to extract the volatile constituents of *G. tenax*. Some compounds were identified by GC-MS (Table 1). These compounds have physiological activity, including antioxidant, anti-inflammatory, and antimicrobial activities. Sesquiterpenes are formed from a 15-carbon skeleton of terpenoids. Widely distributed in nature, the structure of the skeleton is different, and the diverse biological activity of sesquiterpenes has been shown to include antitumor, antibacterial, antiviral, and antimalarial activities [18,19]. Sesquiterpenes can also protect against alcohol-induced gastric mucosal lesions and oxidative damage [20]. (−)-Thujopsene, cedrol, (+)-cuparene, α-curcumene, (−)-β-bisabolene, α-zingiberene are sesquiterpenes were identified. Thujopsene has shown potent antibacterial activity, for example against *Phytophthora ramorum* at 2.0–3.0 ppm [21]. Inhaling vaporized Cedrol ((64.0 ± 7.7) × 10^−9^ M) can affect autonomic nervous functions in humans, such as induce sedative effects, decrease heart rate and blood pressure [22], and (+)-cuparene, α-curcumene and (−)-β-bisabolene mostly exhibit a strong physiological activity [23,24]. Vanillylacetone and fitone both have some physical activity, especially vanillylacetone. For instance, zingerone acts mainly by increasing systemic superoxide dismutase activity to protect the effect of zingerone against 6-hydroxydopamine-induced dopamine reduction, (65 nmol/kg, ip) [25]. Zingerone (10 μg/mL) has also been shown to mitigate radiation-induced mortality and cytogenetic damage, which may be attributed to inhibition of the radiation-induced decline in endogenous antioxidant levels and scavenging of radiation-induced free radicals [26]. Zingerone exerts its potent anti-inflammatory action by increasing HNF-4 and PPAR activities, while suppressing NF-kappaB activity at a dose of either 2 or 8 mg/kg/day for 10 days. [27]. 

### 2.2. Antioxidant Activity Assays

**Figure 2:**
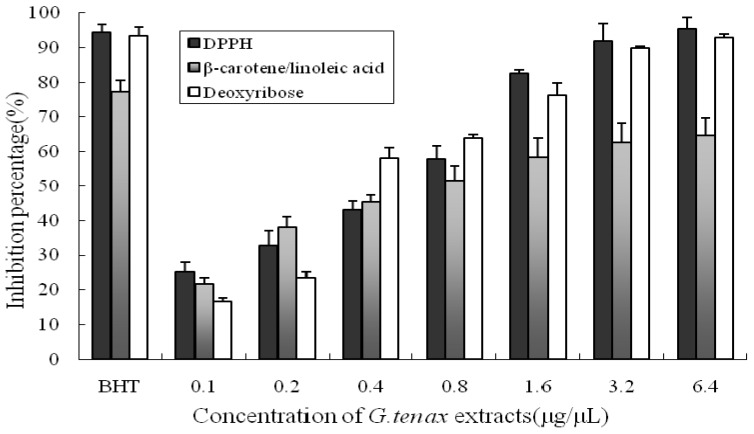
Antioxidant activities of *G. tenax* extract and the positive control as detected by different methods.

Due to the complex nature of phytochemicals and determination of antioxidant activity according to the reaction mechanism, we used three methods to measure the antioxidant properties of *G. tenax* extracts: a 2,2-diphenyl-1-picrylhydrazyl (DPPH) radical scavenging assay, a β-carotene/linoleic acid-coupled oxidation reaction, and a deoxyribose degradation assay. All three methods detected increasing antioxidant activity with increasing dose. Butylated hydroxytoluene (BHT), a strong antioxidant, was used as a positive control and exhibited the highest antioxidant activity. The *G. tenax* extract showed dose-dependent scavenging activity (Figure 2).

Reactive oxygen species (ROS), including hydrogen peroxide (H_2_O_2_), superoxide anion (O_2_^•−^), and hydroxyl radical (OH^•^), are linked to cell damage [28]. Previous literature has demonstrated that the interaction of a potential antioxidant with DPPH depends on its structural conformation. The number of DPPH molecules that are reduced seems to be correlated with the number of electron-donating hydroxyl groups in the antioxidant molecule [29]. This structural requirement could be linked to the presence of phenolic compounds, which are known to be widely distributed in natural herb and spice extracts. Phenolic compounds have a high reducing ability to eliminate free radicals because of both their alcoholic hydroxyl group and conjugated π electrons of the benzene ring [30]. The percentage of inhibition of DPPH by *G. tenax* extracts was evaluated at different extract volumes (Table 2 and Figure 2). In the β-carotene/linoleic acid-coupled oxidation assay, antioxidants are capable of reducing the rate of chain reaction initiated during lipid peroxidation mainly by scavenging the intermediate peroxyl free radicals formed when linoleic acid is oxidized. This also depends on the hydrogen-donating ability of antioxidants [12]. Although hydrogen peroxide itself is not the most reactive, its *in vivo* toxicity can be partly attributed to hydroxyl radical formation in the cells. Addition of hydrogen peroxide to cells in culture can lead to transition metal ion-dependent OH^•^ mediated oxidative DNA damage [31], as well as other detrimental effects on nucleic acids [32], protein [33], and lipids [34]. Deoxyribose on exposure to hydroxyl radicals, generated by Fenton reaction, degrades into fragments and generates a pink chromophore on heating with thiobarbituric acid (TBA) at low pH [35]. We measured the ability of *G. tenax* extracts to scavenge hydroxyl radicals by studying the competition between deoxyribose and *G. tenax* extract for hydroxyl radicals (Table 2 and Figure 2). Phenolic compounds we identified in *G. tenax* extracts, such as p-hydroxybenzaldehyde and 2,2′-methylenebis(6-tert-btyl-4-methylphenol), acted as effective antioxidants and free radical terminators in the deoxyribose degradation assay. Vanillylacetone and fatty acids, the active compounds we identified, may also be related to antioxidant activity of *G. tenax*. Accumulated evidence demonstrates the antioxidant activity of essential fatty acid components extracted from various plants. Therefore, tetradecanoic acid, *n*-hexadecanoic acid, linoleic acid and oleic acid present in *G. tenax* may contribute to the antioxidant activity [36].

**Table 2:** Absorbance of the *G. tenax* extract and the positive control by three different methods: 2,2-diphenyl-1-picrylhydrazyl (DPPH), β-carotene/linoleic acid, and deoxyribose assays.

Sample	DPPH	∆S β-Carotene/Linoleic Acid	Deoxyribose
0.1 μg/μL	0.2206 ± 0.0079 *	0.0154 ± 0.0003 *	0.7544 ± 0.0091 *
0.2 μg/μL	0.1979 ± 0.0124 *	0.0121 ± 0.0006 *	0.6925 ± 0.0159 *
0.4 μg/μL	0.1680 ± 0.0078 *	0.0107 ± 0.0004 *	0.3792 ± 0.0277 *
0.8 μg/μL	0.1237 ± 0.0106 *	0.0095 ± 0.0009 *	0.3255 ± 0.0091 *
1.6 μg/μL	0.0514 ± 0.0030 *	0.0082 ± 0.0011 *	0.2136 ± 0.0299 *
3.2 μg/μL	0.0235 ± 0.0147 *	0.0073 ± 0.0011 *	0.0911 ± 0.0049 *
6.4 μg/μL	0.0134 ± 0.0097 *	0.0069 ± 0.0010 *	0.0628 ± 0.0070 *
BHT (0.05 mM)	0.0162 ± 0.0175 *	0.0044 ± 0.0037 *	0.0623 ± 0.0103 *
Blank	0.2947 ± 0.0074	0.0196 ± 0.0053	0.9365 ± 0.0325

∆S: The difference value of absorbance at 0 min and 120 min in β-carotene/linoleic acid-coupled oxidation reaction; * *P* < 0.05, compared with blank control; The data are expressed as the mean ± SD (*n* = 3).

### 2.3. Antimicrobial Susceptibility Testing

The antimicrobial activity of *G. tenax* was assessed both qualitatively and quantitatively by disc diffusion. *G. tenax* extracts exhibited moderate broad-spectrum antimicrobial action. Zones of inhibition exhibited a dose-dependent relationship with increasing concentrations of extract. Minimum inhibitory concentration (MIC) values for inhibition of *Staphyloccocus aureus*, *Enterococcus faecalis*, *Pseudomonas aeruginosa* and *Escherichia coli* were 3.9, 7.8, 15.6, and 3.9 mg/mL, respectively (Table 3).

**Table 3:** The antibacterial activity of the *G. tenax* extracts by the diameters of the inhibition zones (mm) using the disc diffusion method (minimum inhibitory concentration (MIC) in mg/mL).

Bacterial strains	*G. tenax* extracts dose (mg)					MIC (mg/mL)
	0.3	0.6	1.2	2.5	5.0	
*Staphyloccocus aureus*	24.3	25.1	26.4	27.7	28.9	3.9
*Enterococcus faecalis*	11.2	15.6	20.3	24.5	29.3	7.8
*Pseudomonas aeruginosa*	10.2	15.5	23.1	24.2	26.6	15.6
*Escherichia coli*	15.4	18.9	20.3	21.6	23.2	3.9

Many diseases are caused by microbial infection, indicating the need for more antimicrobial agents [37]. *Gloiopeltis* is often used to treat intestinal diarrhea. Although much is known about the biological activities of *Gloiopeltis* extracts, little is known about the anti-diarrheal mechanisms. *Gloiopeltis* extracts display broad biological effects, such as the ability to regulate the immune system, inhibit bacterial growth, and prevent adhesion of bacteria to target surfaces. We demonstrate that *G. tenax* extracts inhibit bacterial growth, suggesting that *Gloiopeltis* extracts exert their antibacterial activity directly, rather than indirectly by enhancing the immune system.

GC-MS analysis identified compounds, within *G. tenax* extracts, with known antibacterial activity. Sesquiterpenes, such as thujopsene, (+)-cuparene and cedrol, all have antibacterial activity [21,38,39]. Zingerone (vanillylacetone) and its derivatives can inhibit enterotoxigenic *Escherichia coli* heat-labile enterotoxin-induced diarrhea in mice [40]. Studies have shown that zingerone might exert beneficial therapeutic effects on hypermotility-induced diarrhea by abrogating excessive gastrointestinal motility [41]. Therefore, vanillylacetone is the likely active constituent responsible for the antidiarrheal efficacy of *G. tenax*. A number of free fatty acids are known to possess antibacterial activity against Gram-positive bacteria, as well as their esters [42,43]. Some synthetic fatty acid analogs of cholesterol show excellent antibacterial activity *in vitro* [44]. The antimicrobial activity and therapeutic efficacy of oleic acid in a liposomal formulation against methicillin-resistant *S. aureus* is known [45]. The presence of a hydroxyl group in compounds, especially phenolic components, such as 4-hydroxybenzaldehde found in *G. tenax* extract, can destabilize the bacterial membrane to increase the activity of antimicrobials. Such components at low concentrations might be involved in some type of synergism with the other active compounds. 

## 3. Experimental Section

### 3.1. Plant Materials

*G. tenax* was provided and authenticated by the Nan Ao Marine Biological Research Station of Shantou University in Guangdong Province. It was collected at Nan Ao Island. The study area is on the Tropic of Cancer, located 116°56′–117°09′E and 23°23′–23°29′N off the city of Shantou, eastern Guangdong Province, neighboring the Fujian Province, between Hong Kong and Taiwan. This region is at the northern edge of algae distribution in East Asia. 

### 3.2. Chemicals and Reagents

Alkane standard solutions of C8–C20 (mixture No. 04070) and C21–C40 (mixture No.04071) were from Fluka Chemika (Buchs, Switzerland). 2,2-Diphenyl-1-picrylhydazyl (DPPH), β-carotene, butylated hydroxytoluene (BHT), ascorbic acid, and linoleic acid were purchased from Sigma-Aldrich Chemical Co. (St. Louis, MO, USA). 2-Deoxy-D-ribose was purchased from Amresco. 2-Thiobarbituric acid (TBA) and EDTA were obtained from Aladdin (Shanghai, China). All other chemicals and solvents (ethanol, H_2_O_2_, tween-40) were of analytical grade. Ultrapure water was used for the experiments.

### 3.3. Supercritical Fluid Extraction with Carbon Dioxide

CO_2_-SFE involved use of an HA221-50-06 extractor (Huaan Supercritical Equipment Co., Nantong, Jiangsu, China). The instrument was run with CO_2_ for both the extraction and cooling gases. The extraction pressure and temperature were 300 bars and 45 °C, respectively. *G. tenax* powder was placed into 5 L extraction thimbles. The samples were extracted with pure CO_2_, with ethanol used as an entrainer. The amount of ethanol, 1 L, was approximately equal to the sample volume. Fractions were collected in both separators into two separate containers. The addition of a polar co-solvent (ethanol 95%) led to increased efficiency of extraction.

### 3.4. Gas Chromatography-Mass Spectrometry Analysis

GC-MS analysis involved an Agilent 7890 GC equipped with a quadrupole 5975 mass spectrometer (Agilent Technologies, CA, USA) and an HP-5MS column (i.d., 0.25 mm; length, 30 m; film thickness, 0.25 μm). Oven temperature was maintained at 100 °C for 2 min initially and then sequentially raised to 140 °C at a rate of 10 °C/min, to 170 °C at a rate of 1 °C/min, and to 280 °C/min at a rate of 8 °C/min where the temperature was finally held for 10 min. The MS conditions were: ionization voltage, 70 eV; emission current, 10 mAmp; scan rate, 1 scan/s; mass range, 45–450 M/Z; trap temp., 150 °C; transfer line temp., 280 °C. Operating conditions were: injector temp., 280 °C; FID temp., 280 °C.; carrier(He) flow rate, 0.8 mL/min. Samples were injected splitless. Two microliters of sample, dissolved in ethyl acetate was injected. 

Identification of the constituents was based on comparison of their Kovats Index (KI) and the MS fragmentation pattern with reference compositions in the database of the NIST Mass Spectral Search Program (NIST 08 mass spectral database, National Institute of Standards and Technology, Washington, DC, USA). To determine the KI value of the components, a commercial aliphatic hydrocarbon mixture (Sigma-Aldrich) was added to the essential oil before injecting it into the GC/MS equipment and analyzed under the same conditions as above. The following quasi-linear equation for the temperature-programmed Kovats Index was used:

{KI(*x*) = 100 × *Z* + 100 × [*t*(*x*) − *t*(*z*)]/[*t*(*z* + 1) − *t*(*z*)]} (1)

where KI(*x*) is the temperature-programmed Kovats Index of interest and *t*(*z*), *t*(*z* + 1), and *t*(*x*) were the retention times in minutes of the two standard *n*-alkanes containing *z* and *z* + 1 carbons and index of interest, respectively: *t*(*z*) < *t*(*x*) < *t*(*z* + 1).

### 3.5. Antioxidant Assays of Extracts

#### 3.5.1. DPPH Radical-Scavenging System

The DPPH radical scavenging capacity of each herbal extract was evaluated according to Blois, with minor modifications [46]. DPPH radical was prepared in ethanol to a final concentration of 2 × 10^−4^ mol/L. Different amounts of samples at a concentration of 10 mg/mL (solid extract dissolved in 70% ethanol) were added to 150 μL of freshly prepared DPPH radical solution, then 70% ethanol was added to a final volume of 200 μL and the mixture was kept in the dark for 30 min. The absorbance of the reaction mixture was measured at 517 nm. A control was measured in the same way except that the extract was replaced by 70% ethanol. All experiments were performed in triplicate. Scavenging activity (*I*%) was calculated by the equation:

{*I*% = [*A*_control_ − (*A*_sample_ − *A*_blank_)]/*A*_control_ × 100} (2)

where *A*_sample_ is the absorbance of the sample and *A*_control_ is the absorbance of the control. BHT was used as positive control.

#### 3.5.2. β-Carotene/Linoleic Acid-Coupled Oxidation Reaction

Antioxidant activity in the musts was assessed with the β-carotene/linoleate model system [47]. For this purpose, a solution of β-carotene was prepared by dissolving 2 mg in 10 mL of chloroform. An amount of 0.02 mL of linoleic acid and 0.2 mL of tween 40 was subsequently added, and the mixture was left standing at 20 °C for 15 min. After evaporation of the chloroform in a rotary evaporator at 40 °C, 50 mL of oxygen-saturated distilled water at 25 °C was added and the mixture was vortexed vigorously (1 min) to form an emulsion (β-carotene/linoleic acid emulsion). The necessary wells of a 96-well microtiter plate were charged with each different volume of sample and 100 μL of emulsion per well. The microplate was placed on a horizontal shaker and shaken at 100 rpm (during 1 min). A control sample was also prepared in parallel. Absorbance measurements (470 nm) were made at *t* = 0 min and after incubation at 50 °C for 120 min. All experiments were performed in triplicate. Antioxidant activity was expressed as the percent of inhibition with respect to the control sample and calculated as follows:

{AA% = [1 − (*S*_A0_ − *S*_A1_)/(*C*_A0_ − *C*_At_)] × 100} (3)

where *S*_A0_ and *C*_A0_ are the absorbance values of the sample and the control determined at 0 min; the *S*_At_ and *C*_At_ were the absorbance values of test sample and control measured after 120 min. BHT was used as positive control.

#### 3.5.3. Deoxyribose Degradation by Iron-Dependent Hydroxyl Radical

The method of Gutteridge (1987) was employed with minor modifications [48]. Briefly, 1 mL of the reaction solution consisted of the corresponding volume sample, 0.1 mL 1 mM FeCl_3_, 0.1 mL 1.04 mM EDTA, 0.1 mL 20 mM H_2_O_2_, 0.1 mL 2mM L-ascorbic acid and 0.1 mL 60 mM deoxyribose in potassium phosphate buffer (50 mM, pH 7.4). Then, the reaction mixture was incubated for 1 h at 37 °C, after addition of 1 mL of 2.8% (w/v) trichloroacetic acid and 1 mL of 1% (w/w) TBA (1% in 50 mM NaOH). Deoxyribose degradation was measured by the TBA reaction. Further it was heated in a boiling water bath for 15 min and absorbance was measured at 532 nm against a blank. All experiments were carried out in triplicate.

{OH^•^% = [*A*_0_ − (*A*_1_ − *A*_2_)]/*A*_0_ × 100} (4)

where *A*_0_, *A*_1_, *A*_2_ represent the absorbance of control, samples, and blank, respectively. BHT was used as positive control. 

### 3.6. Antimicrobial Susceptibility Testing and Determination of Minimum Inhibitory Concentration (MIC)

Antimicrobial susceptibility testing for *G. tenax* involved the Kirby-Bauer (KB) disk diffusion method against Gram-positive and -negative bacteria. Microorganisms included S*taphylococcus aureus* (American Type Culture Collection (ATCC) No. 25923), *Enterococcus faecalis* (ATCC 29212), *Escherichia coli* (ATCC 25922) and *Pseudomonas aeruginosa* (ATCC 27853). Briefly, a suspension of the tested microorganism (0.1 mL of 10^8^ cells/mL) was spread on solid media plates. Filter paper discs (6 mm in diameter) were impregnated with 10 µL of different concentrations of *G. tenax* extract and placed on the inoculated plates, then incubated at 37 °C for 24 h. 

A broth microdilution method was used to determine the MIC against the susceptible microorganisms using the KB method according to the National Committee for Clinical Laboratory Standards. A serial doubling dilution of *G. tenax* extract from 0.24 to 500 μg/μL was prepared in 50 μL Mueller-Hinton broth (MHB) in each well of a 96-well microtiter plate. Freshly grown microbial suspensions in MHB were standardized to a cell density of 1.5 × 10^8^ (McFarland No. 0.5) and added to the wells (50 μL). After incubation at 37 °C for 24 h, the MIC was defined as the lowest concentration of *G. tenax* extract at which the microorganism did not demonstrate visible growth.

### 3.7. Statistical Analyses

Assays were carried out in triplicate, and each analysis of all samples was performed in duplicate; results displayed are averages. Absorbance values are expressed as means ± SD. Differences between variables were tested by one-way ANOVA with SPSS 13.0 (SPSS Inc., Chicago, IL, USA). Values of *P* < 0.05 were considered statistically significant.

## 4. Conclusions

This study extracted constituents of *G. tenax* through CO_2_-SFE. The results of GC-MS analysis demonstrated that sesquiterpenes, ketones, fatty acids and their esters, phenols, and sterols were the main components of the *G. tenax* extract. Extracts showed remarkable antioxidant activity and moderate broad-spectrum antimicrobial action. Analytical experiments and limited biological assessments indicate that the antioxidant and antimicrobial activities of *G. tenax* might be due to the identified chemical composition. These results suggest that *G. tenax* has potential to be used as a source of natural antioxidants and antimicrobial agents in food processing.
